# Ultrasonography of Normal Adrenal Glands in Adult Holstein–Friesian Cows: A Pilot Study

**DOI:** 10.3390/ani10071171

**Published:** 2020-07-10

**Authors:** J. Daniel Barreiro-Vázquez, Andrés Barreiro-Lois, Marta Miranda

**Affiliations:** 1 Department of Anatomy, Animal Production and Clinical Veterinary Sciences, Faculty of Veterinary Medicine, Universidade de Santiago de Compostela, 27002 Lugo, Spain; josedaniel.barreiro@usc.es (J.D.B.-V.); andres.barreiro@usc.es (A.B.-L.); 2 Veterinary Teaching Hospital “Rof-Codina”, Faculty of Veterinary Medicine, Universidade de Santiago de Compostela, 27002 Lugo, Spain

**Keywords:** adrenal glands, abdominal ultrasound, cow, physiological status

## Abstract

**Simple Summary:**

Knowledge of the normal appearance of the abdominal organs of animals is essential for accurate ultrasound evaluation in daily clinical routine in veterinary practice. In this paper we describe, for the first time, a systematic protocol for ultrasonographic examination of the adrenal glands of cows and provide preliminary reference values for healthy adult Holstein–Friesian cows.

**Abstract:**

Ultrasonographic reference values for the adrenal glands of cattle have not been reported to date. Adrenal glands can be affected by different pathologies, such as hyperplasia, neoplasia and atrophy (either primary or secondary). The present findings indicate that the right adrenal gland can be easily characterized by transabdominal ultrasound in adult Holstein–Friesian cows, with no apparent influence of age or weight. The right adrenal gland (mean length 3.86 ± 1.39 cm; and mean thickness 1.39 ± 0.26 cm) was consistently and mainly located in the 12th intercostal space. The left adrenal gland was more difficult to locate due to its more medial position, and to the presence of gas in the gastrointestinal tract, so it could not be visualized in most animals (18/25). Its mean length was 3.72 ± 0.95 cm, and mean thickness was 1.36 ± 0.33 cm, in the sagittal section. This is the first report of the ultrasonographic appearance of the adrenal glands of cows and of the corresponding reference preliminary values.

## 1. Introduction

Abdominal ultrasound is a useful and routine tool in daily clinical veterinary practice, for small animals and for equines and large ruminants. The ultrasonographic characteristics of the major abdominal organs in cattle have been well described [1], but no description of the adrenal glands examined by B-mode ultrasound has yet been reported.

Adrenal glands are part of many biological mechanisms in the body that influence and are influenced by breeding and production [2]. Adrenal glands are essential for the maintenance of homeostasis during stress, through the hypothalamic-pituitary-adrenocortical axis and the sympatho-adrenomedullary axis [3]. As described for small animals [4], in chronic stress conditions associated in cattle with handling, painful procedures and/or pathologies (including transportation, husbandry system, heat, lameness, cutaneous infections, mastitis, metritis, castration, etc.) [3] can lead to alteration in the size and weight of the adrenal glands [5,6,7].

Some biomarkers of adrenal activity have been established; in this regard Hernández et al. [8] demonstrated that salivary cortisol correlates well with plasma cortisol in dairy cattle, and hair cortisol concentrations have been found to depend on age [6,9]. Although some researchers have correlated adrenal weight and these stress biomarkers, the results are not consistent. Thus, no correlation between adrenal gland weight and hair cortisol concentrations was observed in healthy and diseased (acute and chronic) cows [10], whereas a correlation between chronic pathology and adrenal gland weight and volume has been observed in cows [5].

Naturally occurring hyperadrenocorticism (Cushing’s disease), a common disease in dogs that often affects adrenal integrity, has not been described in cattle. However, the hyperadrenal state has been investigated in normal and experimentally adrenalectomized heifers, in order to determine whether it affects the oestrus cycle, as stress (indicated by the hyperadrenal state) may inhibit progesterone secretion by the corpus luteum [11,12]. More recently, the measurement of plasma cortisol and progesterone levels indicated no correlation between naturally occurring ovarian follicular cysts and adrenal gland function [13].

Other adrenal lesions observed in a post-mortem examination of cows include nodular hyperplasia, medullary cysts, haemorrhage, inflammation, amyloid and neoplasia, which can be primary (e.g., adenoma, adenocarcinoma and pheochromocytoma) or secondary (mainly lymphoma and hemangiosarcoma), and both functional and non-functional [14,15,16,17,18]. Neoplasia is typically observed in older cows with a similar prevalence and types of tumour to those found in small animals and humans (0.71% of the slaughtered cows had cortical nodules or tumours), but are also reported in young animals [14]. Beef cattle are more susceptible than dairy cattle to developing adrenal neoplasms [14]. In a retrospective study of 586 tumours in Brazilian cattle, the adrenal gland was the only endocrine organ affected by neoplasia, accounting for 2.73% of the cases (8 cases of cortical adenocarcinoma and 8 cases of pheochromocytoma). Affected cows ranged from 3–9 years of age, and all cases were incidental findings without clinical significance [16]. Some of these adrenal tumours can mimic typical peripartum pathologies, such as agalactia and puerperal paresis-like syndrome (pheochromocytoma) [14]. Rhabdomyosarcoma, pulmonary carcinoma, fibrosarcoma and mastocytoma metastases have also been described [15,17]. These lesions are typically observed in routine abdominal ultrasound examinations of small animals [4,19], and they are potentially observed in abdominal examinations in cattle.

A single report of a suspected case of primary hypoadrenocorticism (Addison’s disease, AD) has been described in a 4.75-year old Simmental late term pregnant cow with symptoms of colic and ileus [20]. An abdominal ultrasound was performed, but no reference to adrenal glands was included. As reported in dogs and cats, the size of adrenal glands can increase or decrease in cases of hypo- or hyperadrenocorticism, as well as in other endocrinopathies [4,19]; this may also be the case in cattle, but no data have been published until now.

As the location of adrenal glands in cattle does not allow exploration by transrectal ultrasound, a transabdominal window must be used. We have only found one report of the appearance of the adrenal glands determined by a diagnostic imaging technique, computed tomography (CT), in cattle (only calves) [21]. Abdominal CT is a powerful tool, but it is not normally available in the clinical setting, and it is not suited for use in adult large animals. Abdominal ultrasound is therefore considered to be the technique of choice for investigating adrenal pathology in bovine medicine.

The aim of this study is to provide a reference protocol and preliminary values for standard ultrasound exploration of the adrenal glands in cows.

## 2. Materials and Methods

All experiments were performed following Spanish standards for the protection of animals used for scientific purposes. The procedures applied were supervised by the Bioethics Committee of the Rof-Codina Veterinary Teaching Hospital, University of Santiago de Compostela (Spain), and met the criteria for a non-invasive procedure.

### 2.1. Animals and Specimens

Twenty-five healthy, non-lactating, non-pregnant adult Holstein–Friesian cows, used for clinical lectures at the Rof-Codina Veterinary Teaching Hospital, of mean age 6.76 ± 1.06 years and mean weight 559 ± 58 kg, were used in the study. The cows received a daily maintenance ration of hay provided ad libitum and 2 kg of concentrate (given in two equal rations at about 8:00 h and 19:00 h) throughout the study period. Values of haematological, biochemical and urine parameters were normal in all of the cows [22]. The cows were housed in the installations of the Rof-Codina Veterinary Teaching Hospital, University of Santiago de Compostela, Spain.

The right and left adrenal glands excised from two cross breed-Friesian calves (2 months old) were used to correlate in vivo and ex vivo adrenal ultrasound images. They were provided by the Pathology Service of the Rof-Codina Veterinary Teaching Hospital, University of Santiago de Compostela, Spain.

### 2.2. Anatomy

**Figure 1:**
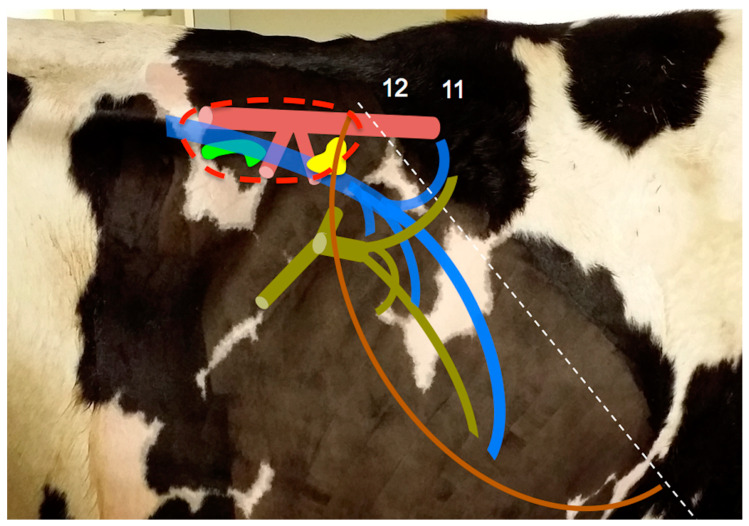
Schematic drawing of the anatomic location of the adrenal glands in the adult cow (sizes have been overrepresented). The right adrenal gland (yellow) can be found in the confluence of the right kidney (dotted red line), caudal vena cava (blue vessels) and the liver (brown line). The left adrenal gland (bright green) is ventral to the aorta (pinkish red) and caudal vena cava. Dark green: portal vein, its main tributaries and portal intrahepatic branches. Numbers indicate specific intercostal spaces. Dotted white line: projection of the caudal margin of the lung.

The right adrenal gland has a typically triangular, “V” or heart-shaped appearance. It is anatomically located adjacent to the caudal vena cava (to which it is attached by connective tissue) at the level of the 12th intercostal space (ICS), between the craniomedial part of the right kidney and visceral surface of the liver (these structures are connected by the hepatorenal ligament). Its ventral surface has a notch for the caudal vena cava that gives the gland the heart or V-shaped appearance (Figure 1). The gland apex points cranially and it is located in the angle between the caudal vena cava and the dorsal margin of the liver [23]. The left adrenal gland is sometimes “C” or comma-shaped, and its position is apparently less constant than that of the right gland, but it is usually displaced to the midline, as other left sided organs in cows, lying ventrally between the aorta and caudal vena cava, at the level of the 1st lumbar vertebra, with its long axis parallel to the sagittal plane of the animal. It is generally located within the perirenal fat, a few centimetres cranial to the left kidney, in close contact with the dorsal curvature of the rumen and immediately caudal to the cranial mesenteric artery. Both glands are flattened (in a latero-medial direction in the right gland, and in dorso-ventral direction in the left gland) and can be subdivided into macroscopically evident cortex and medulla in gross sections. In the anatomic literature, the length of both glands generally varies from 5 to 8 cm, with a mean width of 3–6 cm and 2–3 cm of thickness, with no breed information given. The maximal dimension of each gland, considering its own orientation, corresponds to the dorso-ventral axis for the right adrenal gland, and cranio-caudal axis for the left gland, but this is not well described in the literature. The left gland is typically larger and heavier than the right gland. Blood is supplied to the adrenal glands via the celiac artery (not the aorta itself) [21,23,24,25,26,27].

### 2.3. B-Mode Ultrasonography

As part of a general study of the normal abdominal ultrasound examination in adult cows, a transcutaneous abdominal ultrasound exploration of the right abdomen was performed, from caudal to cranial, as previously described [1,28]. A dorsal approach to the left abdomen was also included, to verify accessibility to the adrenal glands. During the exploration, the cows were standing in stocks without sedation. The hair in the exploration zone was clipped, the skin was washed, and the ultrasound gel was applied, to ensure good contact between the probe and the skin. A B-mode ultrasound examination was performed with a convex array transducer (1–9 MHz) (Esaote MyLab-ClassC^®^, Barcelona, Spain), by the same operator (J.D.B.-V.), always between 17:00 and 19:00 h. Regarding probe’s orientation on the animal; transverse and longitudinal planes were always performed, but difficulties were found obtaining longitudinal planes in the intercostal spaces, due to small space and the large footprint of the convex transducer. Adrenal gland length or long axis and thickness or short axis at its maximal point (perpendicular to each other), as well as echogenicity, were evaluated in each animal. The maximum length and thickness of the adrenal glands were measured at the access point and plane that provided the best images of these organs. Measures were obtained by applying the ultrasound machine software on an image at one point or using two images if the gland could be observed from different approaches (e.g., from the flank and 12th ICS).

An ultrasonographic examination of the adrenal glands dissected from two cross-breed Friesian calves (two months old, obtained from the Pathology Service) with no pathology in the adrenal glands was also performed to correlate in vivo and ex vivo images. The samples were held in a water bath and a linear high frequency transducer (4–13 MHz) was used to examine the glands (Esaote MyLab-ClassC^®^, Barcelona, Spain).

### 2.4. Statistical Analysis

All statistical analyses were conducted using SPSS for Windows v.24 (IBM Corp, Armonk, NY, USA). The normality of the data distribution was checked using the Kolmogorov–Smirnov test. As the data were normally distributed, the results are presented as means and standard deviations. The ranges were calculated as:

(1) $${\bar{X}}_{j\;}-1.96\;SD_{j}\;\mathrm{and}\;{\bar{X}}_{j\;}+1.96\;SD_{j}$$

where ${\bar{X}}_{j}$ is the mean of the variable *j* and $SD_{j}$ is its standard deviation. One-way ANOVA was used to check for possible differences attributable to age and weight. A significance level of *p* < 0.05 was applied in all cases. 

## 3. Results 

As none of the measurements were influenced by age or weight, all cows were included in a single group. 

For the consistent location of the adrenal glands, special focus was given to the dorsal aspect of the cranial abdomen. The large vessels of the abdomen (aorta and caudal vena cava) were used as landmarks.

**Figure 2:**
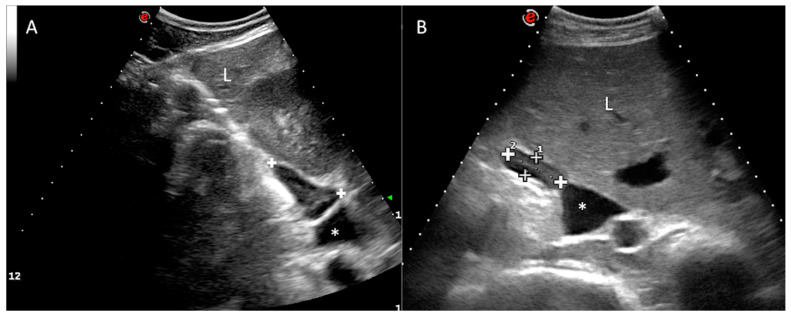
Right adrenal gland. Transverse plane of the abdomen obtained at the level of the 12th right intercostal space, showing the right adrenal gland (between cursors or “+” sign) with an “L” shape (**A**) or elongated shape (**B**). The medulla can be observed as an echoic internal band. Dorsal is to the left of the image. Caudal vena cava (*). Liver (L). Kidney (K).

Regarding the right adrenal gland, a longitudinal plane was not consistently obtained, due to the wide footprint of the convex probe that could not easily fit the dorsal aspect of the intercostal space. However, it was possible to locate the transverse plane of the right adrenal gland (with the transducer aligned to direction of the intercostal space). In all of the cows, the right adrenal gland was clearly visualized at the confluence between the right kidney and the liver, immediately adjacent to the caudal vena cava (Figure 2), from the level of the right flank (7/25) to the 11th ICS (7/25), but mainly in the 12th ICS (14/25), of length 3.86 ± 0.56 cm (range 2.76–4.96) and thickness 1.39 ± 0.26 cm (range 0.90–1.88) in the transverse section. The gland length or long axis corresponds to the dorsoventral dimension, while the gland thickness or short axis corresponds to the lateromedial dimension at its maximal point. The ultrasound image of the right adrenal gland revealed an elongated or “L” shape (Figure 2). The caudal vena cava was the main landmark followed during the exploration. Adrenal architecture is commonly distinguishable in the right adrenal gland, with a thin hyperechoic external capsule, a hypoechoic cortex and an echoic medulla. The parenchyma is smooth and homogenous. The vessels and hilum were not clearly defined. 

**Figure 3:**
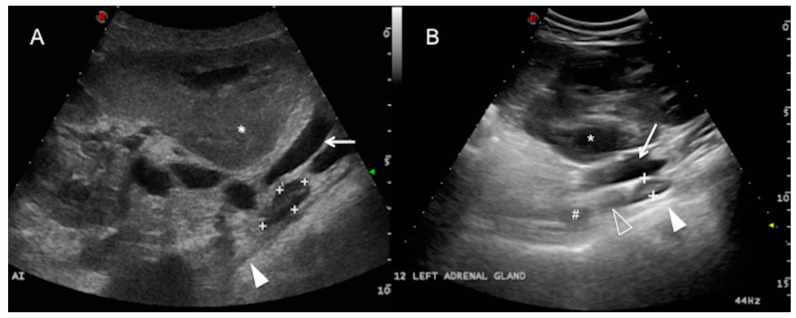
Left adrenal gland. Dorsal plane from the right paralumbar abdomen (**A**) and 12th intercostal space (ICS) (**B**) of two different cows, showing the left adrenal gland (between cursors or “+” sign) adjacent to the aorta (not visible in this plane) and the caudal vena cava (white arrow), medial to the right kidney (asterisk). Note that the gland is in close contact with the dorsal sac of the rumen (white solid arrowhead) and caudal to the coeliac artery (#) and the cranial mesenteric artery (white hollow arrowhead). The adrenal cortex and medulla can be differentiated. Cranial is to the left of the image. AI = left adrenal gland.

The left adrenal gland was only visualized in 7 of the 25 cows examined in this study, ventral to the abdominal aorta and the caudal vena cava from the right side. It was more clearly detected in a longitudinal plane from the flank (3/7) and the 12th ICS (3/7), and it was only visible at the level of the 11th ICS in one cow, caudomedial to the right kidney and caudal to the main arterial aortic branches of the abdomen (coeliac and cranial mesenteric artery) (Figure 3). The left adrenal gland was elongated along the longitudinal plane, sometimes bilobed, and of mean length 3.72 ± 0.95 cm (range 1.86–5.58) and mean thickness 1.36 ± 0.33 cm (range 0.71–2.01). It was not identified in any of the cows from the left abdominal transcutaneous approach. The adrenal cortex and medulla were only discernible in 3 of the 7 cows in which the left adrenal gland was visualized. Adjacent to the gland, a hyperechoic line with acoustic shadowing indicates the presence of the dorsal sac of the rumen. The ruminal wall layers were sometimes identified as a combination of hypo and hyperechoic lines [1] (white solid arrowhead, Figure 3). The main hindrance to identifying the left adrenal gland was interference from the gas in the gastrointestinal tract adjacent to the dorsum of the abdominal cavity (mainly in the rumen and large intestine).

The length and thickness of both adrenal glands were very similar, but the length of the left gland was slightly more variable (ranges of 1.86–5.58 cm and 2.76–4.96 cm for left and right adrenal, respectively).

**Figure 4:**
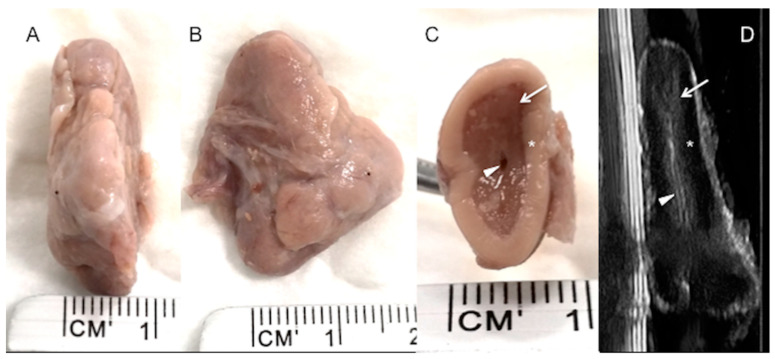
A right adrenal gland from a dead calf. (**A**) transverse view, (**B**) dorsal view and (**C**) cross section at a mid-point. (**D**) corresponds to an ultrasound image through a mid-sagittal cross section of the same adrenal gland held in a water bath, before dissection. Adrenal cortex (*) and medulla (white arrow) are clearly defined in both (**C**,**D**), with the hypoechoic cortex and echoic medulla. Small vessels were identified in this specimen (white arrowhead), but not in the in vivo ultrasound.

High-resolution images were taken of four adrenal glands (two right and two left adrenal glands) dissected from two dead calves, and the adrenal cortex and medulla were clearly depicted (Figure 4). Internal vessels were identified with a high frequency transducer.

## 4. Discussion

Abdominal ultrasound is a non-invasive, non-painful technique that allows investigation of internal structures. It is portable and nowadays affordable for bovine practitioners, although its application requires expert knowledge. Abdominal ultrasound has proved to be useful for detecting adrenal pathology in small animals [4,19] and is considered a primary tool for investigating adrenal pathology. However, no descriptions of adrenal ultrasonography in bovine medicine have yet been published in the scientific literature.

Transrectal ultrasound is the main tool used by vets to examine cows, but clinicians are increasingly extending these skills to the rest of the cow anatomy, including the abdomen, thorax, cardiovascular and musculoskeletal zones. However, other probes are needed in these other situations. For abdominal exploration, a convex low frequency transducer is usually recommended, to reach the deeper parts of the abdominal organs. Nevertheless, although a transrectal probe can also be useful for abdominal exploration, it is limited to superficial structures. Use of a low frequency transducer is recommended for abdominal ultrasonography exploration in cattle.

Our preliminary findings indicate that adrenal glands in adult cows can be located ultrasonographically by a right abdominal/intercostal approach. As in small animal ultrasonography, large abdominal vessels serve as landmarks that can be consistently located in routine ultrasound examinations. For the right adrenal gland, the caudal vena cava at the level of the right kidney and liver is the main landmark, while for the left adrenal gland, it is the caudal vena cava and aorta, caudomedial to the right renal hilum. As with other left-sided organs in cows, the left adrenal gland is displaced to the right of the midline, lying ventrally between the aorta and the caudal vena cava, caudal to the level of the main arterial trunks of the abdominal aorta (the celiac artery and the cranial mesenteric artery), as previously described [24,25,26,27]. The size and shape of the adrenal glands are described, indicating that the right gland is typically heart-shaped and the left is less regular in form and less constant in position. Ultrasonographically, the right gland usually appears as an “L” or elongated shape, because a transverse section is obtained when the ultrasound probe is aligned parallel to the intercostal space. With this approach, the right gland is easily located at the confluence of the right kidney, liver and caudal vena cava. A cranio-caudal or dorsal plane of the right adrenal gland was not obtained, due to difficulties in alignment of the probe within the dorsal aspect of the intercostal space, and the described anatomic heart shape was not observed. This problem is partly but not completely solved by access via the flank because the access point is very dorsal, so that the transverse process of the first lumbar vertebra is also an anatomical obstacle to the correct alignment of the probe at this point. In addition, gas inside the intestinal tract obscures and prevents a good approach to the left adrenal gland. The greater variability in the length of the left adrenal gland than in that of the right gland can be partly explained by the difficulties in obtaining consistently high-quality images of the left adrenal gland.

The division into cortex and medulla is evident in gross sections [26], as it is in the ultrasonographic image described in this study, especially for the right adrenal gland in live animals, and is well correlated with the post-mortem specimens (see Figure 2 and Figure 4). As low frequency transducers were used in this study to reach the adrenal glands, the corticomedullary and vessel resolution were not optimal, especially for the left adrenal gland, which is anatomically deeper. This situation is not suitable for obtaining consistent corticomedullary ratios, as described in the literature, to verify reactivity in different zones of the adrenal cortex or medulla [5,6,29]. We have not included specific measurements for the cortex and medulla, and we cannot correlate our results with those obtained in the study conducted by Blutke et al. [5], in which the increase in size and volume of the adrenal glands can be attributed to a significant selective increase in the zona fasciculata in the adrenal cortex. However, although our preliminary findings confirm that ultrasonographic images can discriminate between the cortex and medulla, small vessels can only be identified in the ex vivo image (Figure 4), but not in the in vivo ultrasound, due to low ultrasonographic frequency used and consequently a worse resolution. 

The appearance of the adrenal glands has been described in one CT study, which provided reference length and width values for healthy calves up to age 105 days [21]. CT has many advantages over ultrasound (i.e., multiplanar reconstruction, absence of overlap of anatomic structures), but it is not available in routine bovine clinical practice and it is not suitable for abdominal examination in adult animals. 

In a more recent paper, Braun [6] obtained different post-mortem measurements on microscopic images for right adrenal width in calves, dividing the gland into capsule, cortex and medulla, and reporting mean values of respectively 0.132 ± 0.022 mm, 1.808 ± 0.268 mm and 3.305 (2.894–3.741) mm. The total estimated thickness of the right adrenal gland in healthy calves was therefore approximately 6.921 mm (6.194–8.201 mm, taking into account the maximum and minimum variations described in the paper). These values were obtained in post-mortem specimens fixed in formalin, and from which tissue sections were cut into rectangular pieces. Although values may therefore not correspond perfectly to in vivo findings, they serve for reference purposes. 

As expected, the adrenal glands (from adult cows) examined in the present study were larger than those (from calves) examined in previous studies. Our data are not strongly correlated with the data reported in a previous book [27]. Only adrenal weight was obtained for adult cows in other different studies [5,10,29], and cannot be compared with our results. Breed and weight are considered variable factors in adrenal gland thickness in dogs [30], and they may also influence the values in cattle. The measurements obtained in the present study only correspond to adult non-lactating Holstein–Friesian cows. In dogs, a ratio comparing adrenal gland size and aortic diameter has recently been used to demonstrate the influence of weight on adrenal size and show the absence of any influence of sex or age on this ratio [31,32]. This should be explored in cattle, by considering different factors such as type of production (beef or dairy), breed, level of production, level of stress, etc. In the present study, we selected a homogeneous group of cows to establish preliminary reference ranges. The thickness of the caudal lobe is the main measurement used to verify adrenal enlargement in dogs [4,19,30]. We observed that the thickness of the adrenal glands was less variable than the length, and therefore length may also be a less significant measurement in cows. It is possible that length is more susceptible to operator error due to misalignment. Thickness may therefore prove to be the main ultrasonographic measurement, although this must be confirmed in future studies. Although a three-dimensional estimation of adrenal volume would be the best option, this is very difficult to achieve by ultrasonography (in contrast to CT).

Several studies have shown that adrenal pathology is more common in cattle than previously thought, and includes primary (mainly adenoma, adenocarcinoma and pheochromocytoma) and secondary tumours (lymphoma as the most common neoplasia), as well as many other typical lesions, such as nodular hyperplasia, haemorrhage and medullary cysts [14,15,16,17,18]. Similarities between cortical adrenal carcinomas in humans and cows have led to bovine carcinomas being proposed as a natural model for human research [17]. Chronic stress, in the form of chronic disease or discomfort, and other endocrinopathies can affect adrenal size in cows and small animals [4,5,10,11,12,19,20]. Such changes in size could be detected by abdominal ultrasound in adult cows, contributing to improving the diagnosis of alterations in a non-invasive and affordable manner.

## 5. Conclusions

A right dorsal approach to the abdomen with a low-frequency ultrasound machine proved a reliable method for characterizing adrenal gland architecture, size and shape, in healthy adult Holstein–Friesian cows, although difficulties were encountered in locating the left adrenal gland. Preliminary reference ranges were obtained for the length and thickness of adrenal glands in healthy adult cows. Further research must be conducted to correlate ultrasound dimensions with real anatomic measures, to clarify the variations in image characteristics and the associations between ultrasound, adrenal pathology, and other metabolic biomarkers.
